# Role of Human Macrophage Polarization in Inflammation during Infectious Diseases

**DOI:** 10.3390/ijms19061801

**Published:** 2018-06-19

**Authors:** Chiraz Atri, Fatma Z. Guerfali, Dhafer Laouini

**Affiliations:** 1 Institut Pasteur de Tunis, LR11IPT02, Laboratory of Transmission, Control and Immunobiology of Infections (LTCII), Tunis-Belvédère 1002, Tunisia; chiraz_atri@yahoo.fr (C.A.); fatma.guerfali@gmail.com (F.Z.G.); 2 Université Tunis El Manar, Tunis 1068, Tunisia; 3 Faculté des Sciences de Bizerte, Université de Carthage, 7021 Jarzouna, Tunisia

**Keywords:** human macrophage, polarization, M1-like/M2-like, inflammation, infectious diseases, leishmaniasis

## Abstract

Experimental models have often been at the origin of immunological paradigms such as the M1/M2 dichotomy following macrophage polarization. However, this clear dichotomy in animal models is not as obvious in humans, and the separating line between M1-like and M2-like macrophages is rather represented by a continuum, where boundaries are still unclear. Indeed, human infectious diseases, are characterized by either a back and forth or often a mixed profile between the pro-inflammatory microenvironment (dominated by interleukin (IL)-1β, IL-6, IL-12, IL-23 and Tumor Necrosis Factor (TNF)-α cytokines) and tissue injury driven by classically activated macrophages (M1-like) and wound healing driven by alternatively activated macrophages (M2-like) in an anti-inflammatory environment (dominated by IL-10, Transforming growth factor (TGF)-β, chemokine ligand (CCL)1, CCL2, CCL17, CCL18, and CCL22). This review brews the complexity of the situation during infectious diseases by stressing on this continuum between M1-like and M2-like extremes. We first discuss the basic biology of macrophage polarization, function, and role in the inflammatory process and its resolution. Secondly, we discuss the relevance of the macrophage polarization continuum during infectious and neglected diseases, and the possibility to interfere with such activation states as a promising therapeutic strategy in the treatment of such diseases.

## 1. Introduction

At the end of the 19th century, Elie Metchnikoff was the first to identify macrophages as large phagocytic mononuclear cells that may play a major role in immunity [1]. Since the discovery of the helper function of T lymphocytes that proved macrophage activation potential [2] and the role played by interferon (IFN)-γ in it [1], macrophages have been described in all tissues of the body, with extremely plastic and heterogeneous phenotypes that adapt according to the tissue and organ in which they reside. Inflammatory diseases, particularly known to use the immune chessboard as a favorite playground, switch on or off regulatory cell functions by modifying intracellular processes or extracellular cytokine signaling, and largely target macrophages. Indeed, the versatile fate and potential of these cells is intrinsically related to the ability of the host to mount an efficient immune response, and are hence the target of many inflammatory processes.

Undeniably, within the framework of immune cells with intricate and complex roles, understanding macrophages remains one of the most challenging tasks. Why are these cells so important? Most studies have established macrophages as an indispensable key player in both innate immune responses and adaptive immunity. They are generally considered as one of the first lines of defense against pathogens, and as such interfere in many immune processes. Interestingly, as an essential support to their antipathogenic role, they have essentially been described as major regulators of inflammatory responses. Indeed, they are implicated in the resolution of inflammation, wound healing, and tissue homeostasis following infection or injury as well as in antigen presentation [3,4,5]. Against danger signals, the innate immune system initiates a protective inflammatory response through different phases, from destroying pathogens and removing cellular debris to repairing tissues and maintaining homeostasis [6]. During the last two decades, the concept of macrophage polarization and its role in inflammation has become increasingly recognized, shedding a new light on the mythical and classical macrophage roles. Macrophage polarization into different phenotypes seems to shape macrophage responses and clusters them according to the stimuli perceived. Depending largely on the diversity of microenvironmental stimuli, the amounts of cytokines produced, and the duration of exposure, macrophages will counteract these pressures by deploying specific faculties, each subset of macrophages showing a particular polarization status. This polarization has been classically clustered into two major macrophage polarization programs, classically activated macrophages or M1 and alternatively activated macrophages or M2, each related to specific immune responses, among which both progression and resolution of inflammation constitutes a critical determinant.

Despite the fact that a wealth of crucial information has already been reported, only a segmented view that hardly recognizes the full value of the biological consequences of macrophage polarization on inflammation has been drawn. In addition, such consequences on humans are generally extrapolated from animal models.

This review aims to summarize principal findings regarding the implication of human polarized macrophages in inflammation and their role in resistance to infection, infectious pathogenesis, and chronic evolution of infectious diseases, with a particular emphasis on their role during leishmaniasis. We argue that understanding the continuum of macrophage polarization, its determinants and effects, and the mechanisms of its fine-tuning would be highly instructive for innovative therapeutic strategies, orienting macrophages towards the most effective polarization status against the disease profile considered.

## 2. Macrophage Polarization and Its Role in Inflammation

Polarization is a complex spatiotemporal field (time and tissue dependent) that implicates intrinsic, extrinsic, and tissue environment stimuli including cytokines, growth factors, fatty acids, prostaglandins, and pathogen-derived molecules. A very basic dichotomic view first classified M1 macrophages as having a proinflammatory phenotype, whereas M2 were considered anti-inflammatory macrophages. However, human studies have shown that both their induction routes and their regulated biological processes do not fall within such a simplistic schema and that the original polarization can be reversible upon environmental changes [7]. In fact, there are many existing macrophage phenotypes that express and produce different molecules. Thus, several studies were interested in identifying markers distinguishing human M1-like and M2-like macrophages, which can be helpful in determining the activation status of human macrophages ex vivo and their potential role in inflammation [8,9]. Indeed, the most commonly related balance to the macrophage M1-like/M2-like axis in humans is the Th1/Th2 paradigm of immune responses [10]. This balance maintains homeostasis, whereas disequilibrium induces chronic inflammation and disease [9].

Macrophage polarization evidence generally corroborates phenotype loss, switch of phenotypes, or phenotype modulation. However, to make things more inextricable, a wide variability of expressed markers for M1 or M2 activation depends on the stimuli, on the cellular substrate (cell lines or primary cells), and on the in vivo or in vitro context [9,11,12,13], whereas murine polarized macrophage subsets are much easier to distinguish, mainly based on nitric oxide synthase 2 and arginase-1 as M1 and M2 markers, respectively. Data from several studies demonstrate that human monocytes are polarized to the M1-like phenotype and then switch to M2-like repair macrophages upon changes in the microenvironmental conditions, and vice versa [14,15]. In addition, M1-like macrophages exposed to IL-13 cytokine showed an M2-like phenotype and gained phagocytic activity, whereas M2-like macrophages that switched to an M1-like phenotype lost their endocytic activity but not their phagocytic activity [9].

In addition to canonical M1-like and M2-like macrophages, regulatory macrophages (MRs) have recently emerged as a large population of cells that play a critical role in limiting inflammation during innate and adaptive immune responses. They produced substantial IL-10 following stimulation with Lipopolysaccharides (LPS) [16] and constitute a novel type of suppressor macrophage implicated in inducing tolerance of solid organ transplants [17] and in the xenoimmune response [18]. Dehydrogenase/reductase 9 (DHRS9) has been considered as a specific marker of this human-specific macrophagic population [19].

Other phenotypes have also been described as being essential in atherosclerosis: (i) The M4 macrophage subset, induced upon C-X-C motif chemokine ligand (CXCL4) stimulation, is characterized by the expression of surface markers such as S100A8, mannose receptor CD206, and matrix metalloproteinase 7, and the release of IL-6 and TNF-α. They are potentially proatherogenic with a weak activation of phagocytosis [20]. (ii) Mhem, as atheroprotective cells, are characterized by heme-dependent activating transcription factor (ATF1) and CD163 expression and participate in hemoglobin clearance via an erythrocyte phagocytosis process. They have increased intracellular iron, reduced oxidative stress, suppressed inflammatory activation, and increased IL-10 production [21,22].

This review will, however, focus only on human M1-like and M2-like polarized macrophages and their roles in inflammation.

### 2.1. M1 Like Macrophages and Inflammation

Classically activated macrophages or M1 macrophages constitute the first line of defense against intracellular pathogens and promote the Th1 polarization of CD4 cells. M1 macrophages occur in an inflammatory environment dominated by Toll-like receptor (TLR) and IFN signaling. In order to induce human macrophage polarization and reproduce these effects in vitro, protocols have used granulocyte/macrophage colony-stimulating factor (GM-CSF) or type II IFN and/or TLR agonists for M1 polarization [23,24,25]. M1-like polarized macrophages exhibited a high level of phagocytic activity, and markers that best characterized them were CD64 and CD80, although the level of expression of these two markers was mainly dependent on the nature of the M1 stimulus (IFN-γ versus LPS versus IFN-γ and LPS) [9]. Transcriptional markers of M1-like polarized cells include IL-12p35, CXCL10, CXCL11, CCL5, and CCR7, among others [26]. As indicated above, M1-like macrophages are characterized by their ability to guide acute inflammatory responses. Indeed, they are able to produce proinflammatory cytokines such as IL-1β, IL-6, IL-12, IL-18 and IL-23, TNF-α, and type I IFN; and several chemokines such as CXCL1, CXCL3, CXCL5, CXCL8, CXCL9, CXCL10, CXCL11, CXCL13, and CXCL16; CCL2, CCL3, CCL4, CCL5, CCL8, CCL15, CCL11, CCL19, and CCL20; as well as CX3CL1; which induce Th1 response activation, facilitate complement-mediated phagocytosis, and type I inflammation [8,11,14,25,27,28]. They are finally characterized by microorganism and matrix debris phagocytosis in the early phases of healing and high antigen presentation capacity [11,14,23,27].

### 2.2. M2 Like Macrophages and Resolution of Inflammation

The M2-like polarized population is particularly involved during parasitic, helminthic, and fungal infections. They are induced in response to Th2 responses. Macrophage colony-stimulating factor (M-CSF), IL-4, IL-10, IL-13, or a combination of these factors [7,8,9,23,24,29] are able to polarize macrophages toward the M2-like phenotype. M2-like macrophages are mainly identified based on the expression of CD64 and CD209, a C-type lectin [9]. Another study has shown that in human tissue, CD163 can be considered as an in situ M2-like marker, but only in combination with the transcription factor CMAF [30]. They are also able to induce IL-13, CCL1, CCL2, CCL13, CCL14, CCL17, CCL18, CCL22, CCL23, CCL24, CCL26, and IL-1R production [9,25]. They can also produce high amounts of IL-8, monocyte chemo-attractant protein-1 (MCP)-1, IP-10, macrophages inflammatory protein (MIP)-1β, and CCL5 or Regulated on Activation, Normal T Cell Expressed and Secreted (RANTES) in order to recruit neutrophils, monocytes, and T lymphocytes in an anti-inflammatory or regulatory response [23,24].

Interestingly, and based on different encountered stimuli, various M2-like macrophages subsets have been described: M2a-like, M2b-like, and M2c-like [11,31]; all of them able to secrete high levels of IL-10 and low or null levels of proinflammatory cytokines such as IL-12 [11,31]. M2a-like macrophages are stimulated by IL-4 or IL-13 and express high levels of CD86 and CD200R and low CD14 and TLR4 levels. They induce IL-10, CCL13, CCL17, and CCL22 production [31,32]. M2b-like macrophages are stimulated by LPS or IL-1β and are characterized by higher CD80 and CD14 expression; IL-10, CCL1, and proinflammatory cytokine production; and lower Human Leucocyte Antigen (HLA)-DR expression and IL-12 secretion [31,32]. M2c-like macrophages are stimulated by IL-10 and produce CCL18 and CCL16. They are characterized by a decrease in CD86 and HLA-DR expression, high CD163, and are involved in preventing tissue inflammation [31,32,33]. Finally, tumor-associated macrophages (TAMs) have been categorized as a novel M2-like subset, namely M2d, which can inhibit proinflammatory M1 macrophages. They are mainly characterized by high IL-10 and low IL-12 and TGF-β cytokine production and CXCL10, CXCL16, and CCL5 chemokine secretion [32]. They constitute the major inflammatory component of the tumoral tissue, contributing to angiogenesis and tumor metastasis [34,35], and can be stimulated by IL-6 and M-CSF.

**Figure 1:**
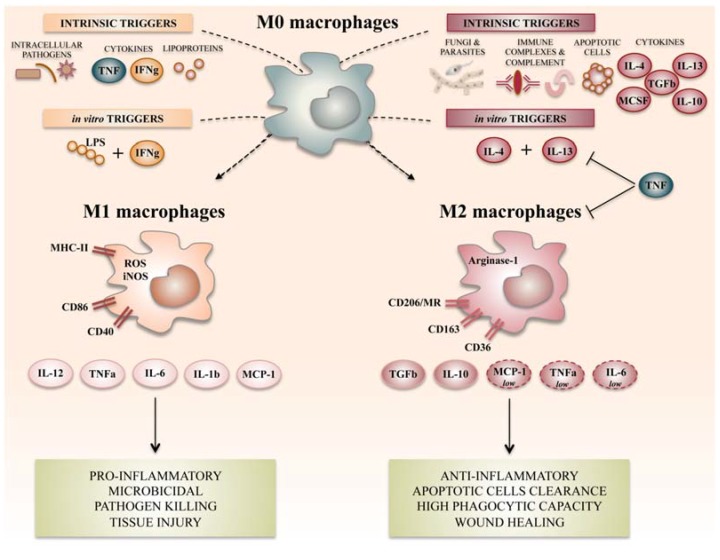
Summary of the main macrophage polarization states of activated macrophages. Different stimuli and signaling pathways have been described as inducers of M1-like or M2-like activation states, of which the most widely referenced ones are summarized here. M1-like or M2-like polarization has been reported in humans as being related to distinct defensive or healing schemas. Many roles have been ascribed to these polarization status, of which pro- and anti-inflammatory macrophage potentiation has for a long time been classically associated to the M1-like/M2-like-like dichotomy. LPS: lipopolysaccharide; MR: mannose receptor; TNF: tumor necrosis factor; IFNg: interferon gamma; IL: interleukin; MCP: monocyte chemoattractant protein; TGF: transforming growth factor; MCSF: macrophage colony stimulating-factor; ROS: reactive oxygen species; iNOS: inducible nitric oxide synthase; MHC: major histocompatibility complex.

Unlike classically activated M1-like, M2-like macrophages have modulator activity (Figure 1), negatively regulate proinflammatory cytokines, and induce production of anti-inflammatory mediators such as IL-4, IL-10, and TGF-β [26,28,36]. Indeed, they are highly endocytic and partially phagocytic, and are involved in a variety of functions including repair mechanisms, homeostasis, metabolic processes, and pathogenesis [9,11].

## 3. M1/M2 Balance, Inflammation, and Its Resolution

It has been acknowledged that the M1/M2 paradigm represents an in vitro extremization of the in vivo setting, in which a “continuum” of activation states exists. In addition, both M1-like and M2-like can undergo reversibly functional changes when exposed to the local cytokine environment that is driving macrophage plasticity and ability to be reprogrammed given the appropriate stimuli [7,9]. This continuum is playing an important dynamic role during inflammation and its resolution.

Inflammation is the physiological response to a variety of injuries, including infections, and leads in this case to a complex immune response characterized by the blood vessel reaction, immune cell recruitment, and release of molecular mediators acting towards pathogen elimination, repairing damaged tissue, and restoring homeostasis. Generally, pathogen recognition by the innate immune system leads to the initiation of the inflammatory cascade and activation of an adequate immune response.

Several studies pointed out the spatiotemporal orchestration of resolving the inflammation process, which can take minutes to a few days for minor damage or over months to years for major damage causing nonresolving inflammation, such as in cancer, inflammatory autoimmune diseases, or chronic inflammation of infection, which may be due to excessive or subnormal inflammatory responses. Hence and accordingly, inflammation can be classified as either acute or chronic. In cancer, inflammation is important for tumor progression. A recent study showed that in lung tumors, M2-like macrophages dominate M1-like macrophages [37]; whereas in colon carcinomas, M1-like macrophages are dominant [38]. It was indeed described in lung cancer that M2a-like and M2c-like macrophages promote cell invasion and tumor progression, whereas M1-like macrophages suppress proliferation, reduce angiogenesis, and induce the apoptosis of lung cancer cells [39]. A type 1 immune response, where macrophages and lymphocytes may play a regulatory and protective role, anti-inflammatory signals suppress inflammation, clear immune cells, and promote remodeling leading to tissue repair, characterizing the acute phase of inflammation, which can persist for several days. Therefore, development of sufficient and adequate anti-inflammatory mechanisms is necessary to suppress the inflammation tissue, promote remodeling, retain homeostasis, and assure the survival of the host [40]. In this context, macrophages, as heterogeneous cells, are fully involved in resolving inflammation at different levels of the inflammatory process. Indeed, they can be the initiators of the inflammatory response and participate in its resolution and maintaining homeostasis in a second step, through the regulation of their own profile polarization upon exogenous or endogenous stimulation and through reprogramming and continuous plasticity.

However, when the inflammatory stimuli is persistent and cannot be destroyed or phagocytized, a subnormal prolonged or excessive inflammatory response or an inadequate production of resolution mediators will lead to tissue damage and chronic inflammation. An imbalance of M1-like and M2-like macrophages may induce in pathological consequences and contribute to several diseases such as asthma [41], chronic obstructive pulmonary disease [42], atherosclerosis [43], or osteoclastogenesis in rheumatoid arthritis patients [44].

During infection, resident tissue macrophages and inflammatory monocytes, recruited from the blood and differentiated into macrophages, induce inflammation to promote pathogen killing. However, macrophage polarization is tightly linked to the processes of resolving inflammation, where the tissue is repaired after infection, but also to nonresolving inflammation, where the pathogen prolongs inflammation. Such polarization might occur at any time during the inflammatory process, and M1-like/M2-like macrophages have different functions that are required to destroy pathogens or repair the inflammation and maintain homeostasis [14].

During the acute phase of inflammation, when first exhibiting a classical M1 activated phenotype, macrophages induce the inflammatory response and release proinflammatory mediators, such as cytokines, chemokines, and reactive oxygen and nitrogen intermediates, which induce the activation of various antimicrobial mechanisms that contribute to pathogen killing and inflammation resolving [10].

However, such responses must be controlled to prevent tissue damage to the host and avoid severe immunopathologies through the production by M2-polarized macrophages of anti-inflammatory cytokine mediators, cytokines, and chemokines that will regulate M1 macrophages, extenuate inflammatory reactions, and promote and accelerate the wound healing process and tissue repair [14,45]. Such a fine-tuned balance and switching back and forth between the M1 and M2 polarization states is necessary to allow the beneficial processes of stress, inflammation, resolution, and repair.

## 4. Swinging Macrophage Polarization During Infections

To circumvent invaders, macrophages are generally polarized toward the M1 phenotype that possesses bactericidal activity to ensure a protective role during the early stages of the infection. As such, pathogens have developed, in turn, several mechanisms to evade the immune response and ensure their survival and replication and disease progression. Indeed, it is well known that several mechanisms are employed by viral, parasitic, fungal, and bacterial pathogens to induce macrophage deviation and ensure their evasion from the host immune response. They hence inhibit the inflammatory cytokine production mediated by pattern recognition receptors/sensors (PRRs) and the polarization of macrophages toward the M1 phenotype in order to decrease the proinflammatory response and activate the M2-polarized macrophages [46]. Once again, this strictly clear-cut dichotomy is not always observed during infections, and pathogens are always acting toward “tailored” inflammation.

### 4.1. Bacterial and Fungal Infections

Upon bacterial infection, macrophages utilize their PRRs to identify pathogen-associated molecular patterns (PAMPs) and endogenous danger signals such as danger-associated molecular patterns (DAMPs). Pathogen recognition by PRRs (i.e., TLRs) activates macrophages to produce M1-like pro-inflammatory mediators including cytokines such as TNF-α, IL-1β, IL-6, IL-12, and NO that kill these invading pathogens. However, and as indicated earlier, some intracellular bacterial pathogens have developed sophisticated strategies to prevent M1-like polarization, altering microbicidal mechanisms or driving the polarization toward an M2 phenotype to reduce the inflammatory response.

*Mycobacterium* (*M.*) *tuberculosis* acute infection induces macrophage polarization toward the M1 phenotype, which secretes high amounts of proinflammatory mediators such as TNF-α, IL-6, IL-12, CCL5, and CXCL18. A study conducted on the THP1 human cell line showed that Rev-erbα, a nuclear receptor, represses IL-10 production, thus providing microbicidal macrophage properties and reducing proliferation of the intracellular *M. tuberculosis* [47]. To counteract such a response, it has been shown that these intracellular bacteria have the ability to inhibit the transcription of IFN-γ target genes, a cytokine implicated in M1 polarization [48] and contributing to the development of tuberculosis pleural effusion [49]. However, polarization of granulomatous macrophages co-expressing proinflammatory and anti-inflammatory markers in the lung of *M. tuberculosis*-infected patients has been described, indicating that polarization is not binary, but occurs along a spectrum [50]. This observation is corroborated by results indicating that the activation state of macrophages underwent M1-like to M2-like transition during the formation and development of tuberculous granulomas [51].

*Helicobacter* (*H.*) *pylori*, the causative agent of chronic gastritis, ulcer disease, and some other gastric cancers, has been described to induce an enhanced M1-like phenotype in gastric macrophages of patients with atrophic gastritis [52], whereas mixed M1-like/M2-like macrophages have been detected in biopsies from gastric mucosa of *H. pylori*-infected patients [53]. It was also shown that during *Salmonella* (*S.*) *typhimurium* infection, intermediate host functional states between the M1-like and M2-like extremes can be observed [54]. In addition, an M1-like/M2-like signature transition has been also observed during treatment or convalescence of patients infected with *S. typhi* [55]. These observations clearly indicate again that the M1-like/M2-like dichotomy is not yet clearly defined in humans and is intimately linked to the inflammatory tissue microenvironment.

Several other pathogens also tend to drive the differentiation of macrophages into the M2-like phenotype and inhibit the inflammatory response through diverse mechanisms. *Candida* (*C.*) *albicans* is the most important opportunistic fungus in nosocomial infections. To circumvent immune responses and enhance its survival and colonization, *C. albicans* induces an M1-like to M2-like switch that reduces inflammation [56] by blocking NO production in macrophages through induction of host arginase activity [57], among other pathways.

### 4.2. Viral Infections

Similarly to bacteria, macrophages are also targeted by viruses and their polarization is manipulated during viral infections. This pathogen manipulation is still not clear and reflects a continuum of different polarization states. Generally, it is accepted that directly after virus recognition, macrophages endocytose the invading pathogens, polarize to the M1-like phenotype [58], and present viral peptides via the major histocompatibility complex (MHC) to T lymphocytes that will then produce IFN-γ and other molecules to recruit effector immune cells and induce an inflammatory response capable of controlling viral replication. A polarization toward M2-like is then established to circumvent inflammation excess within the damaged tissues. However, and although elevated IL-10 expression during hepatitis B virus (HBV) and hepatitis C virus (HCV) infections have been demonstrated to promote an anti-inflammatory phenotype [59,60], other studies showed that HCV inhibits monocyte differentiation to either M1-like or M2-like macrophages through TLR2, associated with an impaired signal transducer and activator of transcription protein (STAT) signaling pathway [61], or induces a mixed M1-like/M2-like cytokine profile [62], leading to dysfunctions of both M1-like and M2-like macrophages in chronic HCV-infected patients, although there is a significant increase in M2-like circulating monocytes in these patients [62]. Interestingly, H3N2 influenza virus subtype-infection induces comparable TNF-α, IL-12, IP-10, and IL-6 levels in both M1-like and M2-like monocytes and macrophages, and both macrophage types could be readily infected with this virus; an observation in marked contrast with the clearly distinct cytokine profiles of M1-like and M2-like macrophages exposed to bacterial products [63]. When analyzing the whole transcriptome of macrophages infected with highly pathogenic H5N1 influenza virus, another study showed that the activation state of the macrophage population is variable across time, suggesting that macrophage subtype switching is a highly plastic process [64].

Human cytomegalovirus (HCMV) is a typical example of manipulating the balance between M1-like and M2-like polarization according to the infectious process. Since a proinflammatory state provides the tools to drive infected monocytes from the blood into the tissue to promote viral dissemination during the early infection stage, HCMV induces infected monocytes to display a unique M1-like/M2-like polarization signature that is skewed toward the classical M1-like activation phenotype [65] and increases secretion of proinflammatory cytokines and chemokines, including IL-1β, IL-6, and TNF-α [66]. However, and during the late phase of infection, viral IL-10 is driving infected cells toward M2-like polarization, which may limit virus clearance by restricting proinflammatory and CD4 T cell responses at sites of infection [67]. In addition, M2-like polarized macrophages show high permissiveness for HCMV infection and optimal susceptibility in comparison to M1-like cells [68]. Macrophage polarization to M1-like and M2-like cells is also important to macrophage susceptibility to HIV infection and replication. Indeed, changes in macrophage polarization represent a mechanism used by macrophages to swing between resistance or latent infection and productive viral infection and spreading [69]. A model starting with M1-like macrophages with accelerated formation of viral reservoirs in a context of Th1 and proinflammatory cytokines (dominated by chemokines production including CCL3, CCL4, and CCL5; ligands of CCR5, the main HIV entry receptor), then with IL-4/IL-13 alternatively activated M2-like macrophages (dominated by IL-10 and CCL18 production) that will stop the expansion of the HIV-1 reservoir has been proposed [70]. An IL-10 deactivation of macrophages will finally lead to immune failure observed at the very late stages of the HIV-1 infection and contribute to the establishment of the chronic activation determinant of HIV disease progression [70].

### 4.3. Parasitic Infections

Both M1-like and M2-like macrophage subsets are involved in parasitic infections; macrophages undergo, in general, a dynamic switch toward the M2-like phenotype at later stages [71]. Given the putative anti-inflammatory effects of helminth *Ascaris suum* antigens in mice, studies have been conducted on human cells and showed that such antigens appear to exert stronger activity when acting upon macrophages that have already been polarized to the M1-like phenotype, rather than influencing the polarization process per se [72]. However, very few studies conducted on human patients or cells have been conducted regarding the in vivo and/or ex vivo effect of parasites on macrophage polarization. This scarcity cannot allow, as for other pathogens, a conclusion to be reached regarding the continuum of plasticity of macrophages during these diseases. The example of leishmaniasis is indeed very instructive.

### 4.4. Leishmaniasis

*Leishmania* (*L.*) parasites are obligate intracellular pathogens that preferentially invade macrophages where they replicate, ultimately causing a heterogeneous group of diseases with cutaneous or visceral manifestations [73] that affects millions of people, mainly in subarid, tropical, and subtropical areas. The form and severity of the disease depend on the infecting *Leishmania* species, the phlebotomine vector species, and also on the immune status of the infected individuals [74].

Strikingly, macrophages play a dual function in infection, acting as a safe shelter for parasites, but also as their ultimate killer, making such cells the alpha and the omega of host resistance or susceptibility to *Leishmania* infection. Similarly to several other pathogens, *Leishmania* has developed several mechanisms to inhibit the immune antiparasitic response, resulting in the appearance of different clinical signs of these infections [75]. Several seminal studies have shown that development of Th1 responses induces parasite killing and resistance to leishmaniasis, while susceptibility to infection is related to Th2 development, parasite replication, and persistence [76]. We previously showed that several genes encoding proinflammatory mediators (e.g., S100A10 and S100A11) were upregulated, while other family members (e.g., interferon gamma receptor (IFNGR)2; STAT1; and interferon regulatory factor (IRF)1, S100A6, S100A8, and S100A9) were downmodulated in human macrophages infected with *L. major* parasites, the causative agent of cutaneous leishmaniasis [77]. Hence, macrophage polarization is critically important during *Leishmania* infection because of the protective and pathogenic functions of macrophages, mediating intracellular parasite killing and controlling tissue damage and repair [78]. Indeed, classically activated M1-like macrophages show leishmanicidal activity, whereas alternatively activated M2-like macrophages exhibit anti-inflammatory activity and favor parasite survival [79,80].

Both M1-like and M2-like macrophages are induced during *Leishmania* infection. Indeed, development of a Th1 response within the course of the disease induces production of proinflammatory cytokines such as TNF-α, IL-12, and IFN-γ and M1 macrophage polarization that leads to NO release, which play a crucial role in the protective immunity against *Leishmania* through parasite killing. In contrast, a Th2 response induces anti-inflammatory cytokine production such as IL-4 and IL-13 and stimulates M2-like macrophage polarization to produce arginase, leading to the inhibition of inflammation, parasite survival, and disease progression [81].

Although several studies analyzed the effect of *Leishmania* parasites on macrophage polarization in well-developed experimental models or naturally infected animals, showing that M2 macrophages can impede protective immunity to protozoan infection, we will describe only the state of the knowledge in human studies.

An in vitro study has evaluated whether polarization of human macrophages (primary and cell lines) to M1-like and M2-like polarized cells has similar effects on their ability to sustain *Leishmania* infection. Authors showed that parasite infection can knock down selected non-coding (nc) RNAs in their host cells through the induction of degradation of a specific RNA polymerase III transcription factor subunit, TFIIIC110, specifically in alternatively activated M2 macrophages [82]. Parasite surface protease gp63 and lipophosphoglycan (LPG) of the promastigote forms, both described as virulence factors, seem to be responsible for such an effect. Such a mechanism might result in parasite survival and inhibition of inflammation during leishmaniasis [82].

**Figure 2:**
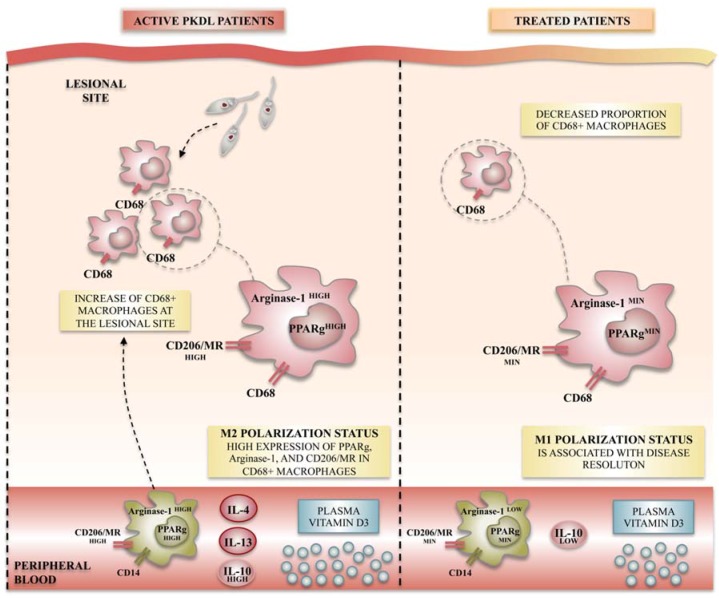
Macrophage polarization status observed in post-kala-azar dermal leishmaniasis (PKDL) patients during acute disease and after treatment. *Leishmania* infection in humans has been studied in PKDL patients, in which features of M2-like polarization have been observed in both lesion sites and peripheral blood. M2-like polarization is characterized by the increased mRNA and/or protein expression of specific M2-like markers such as the nuclear peroxisome proliferator activated receptor γ (PPARγ), the arginase-1 receptor, and the membrane mannose receptor CD206/mannose receptor (MR). After treatment, disease resolution is characterized by an M1-like profile repolarization, evidenced by the decreased expression of M2-like markers.

It is well accepted that differentiation of macrophages toward M1-like or M2-like macrophages is mainly regulated by genes including iNOS, arginase-1, and mannose receptor (CD206), among many others (Figure 2). In this context, Mukhopadhyay et al. studied the functional polarization of monocytes during the post-kala-azar dermal leishmaniasis (PKDL), the dermal sequel of visceral leishmaniasis (VL). They demonstrated that monocytes obtained from PKDL patients showed a decreased expression of M1-like macrophage markers such as TLR-2/4 and NO, whereas an increased expression of M2 markers such as CD206 and arginase-1 were noticed. Additionally, the levels of IL-4, IL-10, and IL-13, an environment favorable to M2-like polarization, were significantly elevated compared to controls. Finally, levels of vitamin D, the signaling of which has been classically linked to M2 polarization, rise during PKDL. Such skewing toward the M2 phenotype likely ensures parasite survival [83]. This study provides the first characterization of M2-like polarized macrophages in human dermal leishmaniasis. More interestingly, when applying antileishmanial chemotherapy, the same authors demonstrated a repolarization of monocytes toward the M1-like phenotype, with reduced amounts of most components of the vitamin D signaling pathway, suggesting that therapeutic approaches to restore the M1-like versus M2-like balance may be effective for disease cure [83].

More recently, and after showing that splenic macrophages in experimental VL demonstrate a mixed activation phenotype, with IFN-γ inducing proteins paradoxically more permissive to parasite replication and growth, Kong et al. suggested that such phenomena could be extrapolated to some human patients suffering from VL [84]. They interestingly hypothesize that the chronic inflammatory environment in the spleen conditions macrophages in VL, and that IFN-γ, as part of the splenic proinflammatory response, drives an exuberant STAT3-induced response that promotes disease and parasite growth [84].

## 5. Conclusions

As highlighted here, there is evidence that this differential polarization of macrophages in diverse infectious disease conditions demonstrates the plasticity of these cells, with M1-like polarization evident in inflammatory diseases, whereas an M2-like polarization has been proposed in chronic parasitic, viral, or bacterial diseases. However, macrophage polarization phenotypes are not always mutually exclusive, and it still remains unsolved if some functional subsets represent real distinct populations or subtle variations in proinflammatory and anti-inflammatory macrophages. Strategies of reshaping macrophage polarization and inflammation could be a promising therapeutic modality worthy of future consideration in several infectious diseases.
